# FACTORS INFLUENCING CANCER SURVIVORSHIP AMONG NIGERIAN WOMEN

**DOI:** 10.1093/geroni/igac059.2237

**Published:** 2022-12-20

**Authors:** Darlingtina Esiaka, Candidus Nwakasi, Runcie Chidebe, Khadijat Banwo Fatai, Gloria Orji

**Affiliations:** Robert Wood Johnson Medical School, Rutgers University, Newark, New Jersey, United States; Providence College, Providence, Rhode Island, United States; Health and Psychological Trust Center, Abuja, Federal Capital Territory, Nigeria; Project PinkBlue, Abuja, Federal Capital Territory, Nigeria; Network of People Impacted by Cancer in Nigera, Abuja, Federal Capital Territory, Nigeria

## Abstract

Breast cancer is the most common cancer globally and among Nigerian women. With technological and medical advancements, especially in the early detection of chronic illnesses, there is a reduction in cancer deaths, and more women are living as cancer survivors in Nigeria. Despite the increase in the number of cancer survivors, many survivors struggle with life after cancer diagnoses. In this study, we interviewed 30 Nigerian women (Mage = 42.00) who completed cancer therapy in the past 12 months and above, to explore how women who underwent cancer therapy perceive their healing, health, and quality of life. An example of one of the interview questions is “How did cancer diagnoses affect your normal way of life?” Each interview lasted an average of 1 hour. Participant responses were transcribed, and theme coded for analysis. We found that fear of reoccurrence, loss of sense of purpose, shame from physical mutilation related to mastectomy, poor psychological health, social isolation, and ill-treatment from other members of the community were recurring themes across all of the women’s responses. The presence of cancer survivors' support groups and religiosity were important for the experience of life after a cancer diagnosis. Our findings draw attention to the need for interventions targeting cancer survivors. The result further indicates that addressing community perceptions of breast cancer is imperative to ensure the health of breast cancer survivors, especially in low-resource countries.

